# Comparing porcine versus bovine mitral valve replacement in terms of structural valve deterioration: a systematic review

**DOI:** 10.1186/s13019-025-03686-2

**Published:** 2025-11-26

**Authors:** Sten Kajitani, Wesley Chorney, Ahmad Albakri, Karolina Kmieciak, Mpho Mthethwa, Martin Ho, Anthony Goodings, Hiroki Harada, David Rabkin, Michio Kajitani

**Affiliations:** 1 School of Medicine, University College, College Rd, Cork, Ireland; 2 Cottage Hospital, Santa Barbara, CA, USA; 3 Takatsuki General Hospital, Osaka, Japan; 4 Cardiothoracic Surgery Department, Loma Linda, CA, USA; 5 Cardiothoracic Surgery Department, St Bernardine Medical Center, San Bernardino, USA

## Abstract

**Background:**

Bioprosthetic mitral valve replacements (MVR) commonly utilize either bovine or porcine valves; however, in terms of structural valve deterioration (SVD), clinical superiority between these valve types remains controversial. The primary objective of this study was to directly compare structural valve deterioration between porcine and bovine bioprosthetic valves used in mitral valve replacement.

**Methods:**

A systematic review was conducted following PRISMA guidelines. Studies directly comparing porcine and bovine bioprosthetic valves in MVR were identified through comprehensive searches of Embase, MEDLINE, and Web of Science databases from inception through March 5, 2025. Eligible studies were cohort studies that directly compared bovine and porcine bioprosthetic valves implanted in the mitral position and reported SVD outcomes. The primary exposure was valve type, and the primary outcomes were reports of SVD. Data extraction included patient demographics, valve characteristics, SVD definitions, and modes of SVD. The Newcastle-Ottawa Scale was used for risk-of-bias assessment. Principal analyses involved narrative synthesis.

**Results:**

Nine studies comprising 6,945 patients (range per study: 240–1,695) with follow-up periods ranging from 3.5 to 15 years were included. Three studies favored porcine valves, two favored bovine valves, and four showed no significant difference in terms of SVD. Porcine valves frequently demonstrated leaflet tearing resulting in acute regurgitation, while bovine valves predominantly exhibited calcification leading to stenosis. Younger patients (< 65 years) generally showed better results with porcine valves. Despite variability across studies, cumulative evidence suggested a trend toward superior long-term durability of porcine bioprostheses.

**Conclusions:**

Valve selection should be tailored to patient-specific factors, including age, anticipated longevity, clinical risk profile, and religious preference. Future studies should employ standardized definitions and longitudinal follow-up to clarify these findings.

**Supplementary Information:**

The online version contains supplementary material available at 10.1186/s13019-025-03686-2.

## Introduction

Valvular heart disease contributes significantly to morbidity and mortality, often leading to heart failure, recurrent hospitalizations, and other cardiovascular complications [1, 2]. Mechanical valve replacement is a consideration for many with valvular heart disease though it carries many side effects, including an increased risk of developing thromboembolism and a necessity for lifelong anticoagulation [3]. Bioprosthetic valve replacement offers an attractive therapeutic alternative by improving hemodynamic performance and symptom relief for those with valvular disease while eliminating the need for lifelong anticoagulation, a critical benefit for patients in whom such therapy is contraindicated, including the elderly (particularly those aged 70 and above) [4].

However, the main set-back of bioprosthetic valves is their tendency to develop structural valve deterioration (SVD); an acquired intrinsic deterioration of the valve structure resulting in hemodynamic dysfunction and often requiring reoperation [5]. In mitral valve replacement (MVR), bioprosthetic options are predominated by bovine and porcine valves. Although bovine valves are commonly used in aortic positions due to the robust durability of bovine pericardial tissue [6], there are some concerns with their use in the mitral position. In particular, their increased structural rigidity may predispose them to earlier SVD. However, porcine valves, with their enhanced leaflet mobility, may demonstrate lower rates of SVD and reduced reoperation frequencies [7]. These biomechanical differences underscore the need for a focused evaluation of valve performance in the mitral setting.

Current clinical guidelines add complexity to prosthetic valve selection. Both the ESC/EACTS 2017 and 2020 ACC/AHA guidelines for the management of valvular heart disease treat bovine and porcine valves as equivalent options [8, 9]; however, this notion is contradicted by recent research directly comparing porcine and bovine valve replacement in the mitral position [10–14]. Moreover, the contradictory findings between some of these studies, as well as other studies concluding no significant differences between the valves [15–18], highlights a critical gap in our understanding of their relative performance in MVR.

Previous reviews have often drawn indirect comparisons by combining studies that report on either bovine or porcine valves independently. For example, the 2019 meta-analysis by Malvindi et al. analyzed over 15,000 patients across 40 studies and concluded that newer-generation porcine valves, particularly the Mosaic prosthesis, offered superior survival and lower SVD rates compared to earlier porcine models and bovine pericardial valves [19]. Despite its comprehensive scope, this study was limited by its indirect comparisons. Similarly, the 2024 meta-analysis by Koulouroudias et al. focused on survival, SVD, and reoperation rates but was restricted to post-2010 studies, thereby excluding valuable historical data. In addition, it employed non-uniform SVD definitions and relied on reconstructed patient data from Kaplan-Meier curves, methods that may not fully account for individual patient variability and unreported confounders, with a limited sample size when comparing bovine and porcine MVRs, fewer than 3000 participants, further reducing generalizability [20]. Additionally, though the specific method used to reconstruct patient data from the Kaplan-Meier curves, IPDfromKM, has a high accuracy and low root-mean-square error, it produces estimates, which are not as desirable as actual patient data [21].

Our systematic review’s aim is to address these previous gaps by exclusively incorporating studies that directly compare bovine to porcine bioprosthetic valves in MVR. By focusing solely on direct comparisons conducted within similar surgical contexts, we aim to minimize confounding factors such as variations in surgical contexts, surgeon expertise, and patient demographics. Ultimately this systematic review’s objective is to directly compare porcine and bovine bioprosthetic valves in terms of structural valve deterioration following mitral valve replacement, providing evidence-based guidance to optimize valve selection in clinical practice.

## Methods

The registered record of this review’s protocol is available with PROSPERO, no. CRD42023475188 [22].

### Search strategy

A comprehensive search strategy was implemented to identify all pertinent studies. Databases included Embase via Elsevier, MEDLINE via Ebsco, and Web of Science (WOS). The search strings were constructed by SK and MK, and validated by a librarian (VC). Search strings for Embase and Medline employed respective Emtree or MeSH headings as well as synonyms for subject terms found in the titles and abstracts, while search strings through WOS employed synonyms for subject terms by topic. On Mar 05, 2025, a search with the fields: “Mitral Valve Replacement,“AND (“Bovine Valve Bioprosthesis,” **OR** “Porcine Valve Bioprosthesis”) was conducted from inception to Mar 25, 2025 (Supplemental Material 1).

## Screening, data Extraction, and risk of bias

The review aimed to include all relevant research on bioprosthetic MVR directly comparing porcine and bovine valves. Eligibility criteria comprised randomized controlled trials and observational cohort or case-control studies enrolling adult patients undergoing porcine versus bovine pericardial bioprosthetic MVR, and reporting SVD outcomes. SVD was defined as intrinsic valve failure (e.g., calcification, leaflet tear, stent creep, or suture disruption) resulting in stenosis or regurgitation, with or without reoperation. Inclusion and exclusion criteria are detailed in Supplemental Material 2.

Studies were excluded if they were in vitro, animal, cadaver, or pediatric studies; involved valves in positions other than the mitral position; used transcatheter, mechanical, or double valve replacements; consolidated outcomes for aortic and mitral valves; or reported SVD due to infection or thrombosis. Editorials, commentaries, and studies lacking direct comparisons between porcine and bovine MVR were also excluded. We did not exclude studies based on design (e.g., non-cohort) or language (e.g., non-English) to minimize potential selection bias. All studies meeting the core eligibility criteria were considered at full-text screening, including those published in other languages, with translation when necessary.

The screening and selection process followed the PRISMA guidelines. The search results were screened by title and abstract by AG and KK. Full texts of eligible articles after the initial screening were retrieved by SK and WC and screened by MH and AA. Discrepancies in the title and abstract or full text screening process were resolved through consensus among the senior authors (MK and DR). Studies were analyzed for Risk of Bias (ROB) using the Newcastle Ottawa Scale (NOS) by HH and MM [23]. Data Extraction was completed by MH and AA using a piloted standardized form. Extracted data included population demographics, valve type, including manufacturer and MVR model, SVD definitions, and the modes/mechanisms of SVD. Primary outcomes extracted included time to SVD, time to reoperation for SVD, incidence of SVD, incidence of reoperation for SVD, risks of the development of SVD, freedom from SVD, freedom from reoperation for SVD, explant probability for SVD, and valve survival. Given the heterogeneity of the included studies’ reported outcomes, a narrative synthesis was utilized to summarize the findings. Because all included studies compared porcine and bovine bioprosthetic mitral valves within the same study population, baseline characteristics were generally assumed to be comparable. In concurrent single-center series, this assumption is strong; in multi-center or era-separated studies, residual confounding from differences in surgical teams, patient selection, and institutional protocols cannot be excluded. Where available, reported methods such as propensity score matching, propensity score–adjusted multivariable analysis, Cox regression analysis, Cox proportional hazards analysis, inverse probability weighting, or competing-risks regression to address baseline imbalances were noted.

## Results

### Summary of the results

**Fig. 1:**
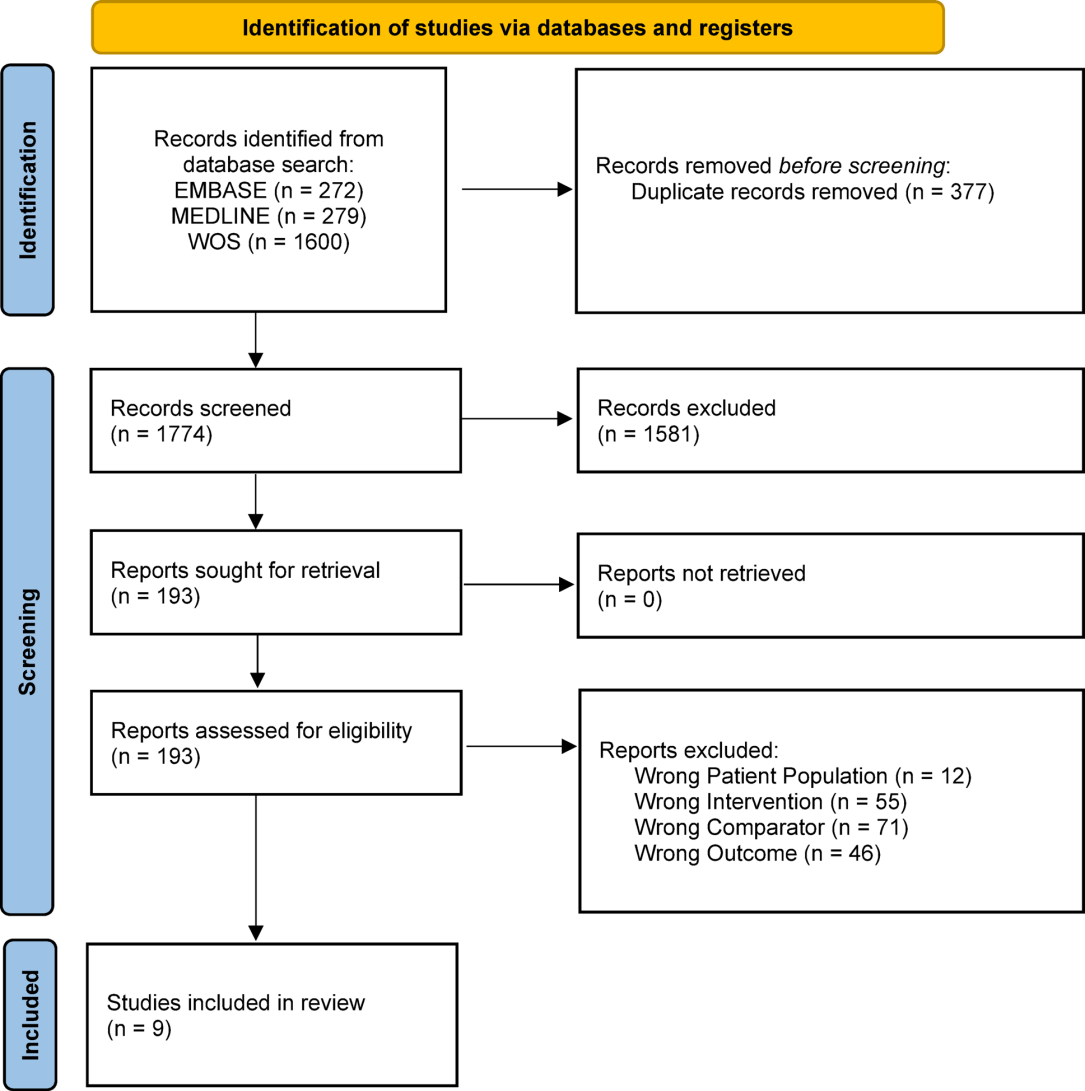
Prisma Flow Diagram: The initial search yielded 2,151 records from Embase, MEDLINE, and Web of Science. After removing 337 duplicates, 1,774 records were screened by title and abstract. Of these, 1,581 were excluded for not meeting eligibility criteria (e.g., unrelated to mitral valve replacement, not involving both porcine and bovine valves, not reporting structural valve deterioration [SVD] outcomes). A total of 193 full-text articles were assessed for eligibility, of which 184 were excluded for the following reasons: 12 were vitro/animal/cadaver or pediatric studies; 55 studied valves in positions other than the mitral position; transcatheter, mechanical, or double valve replacements; 71 combined aortic and mitral outcome or lacked direct comparison between porcine and bovine MVRs; and 46 studies’ SVD outcomes were due to infection or thrombosis. Nine studies met all inclusion criteria and were included in the final review

Nine studies involving 6,945 patients with follow-up durations ranging from 3.5 to 15 years were included in this systematic review (Fig. 1; Tables 1 and 2, Supplemental Material 3) [10–18]. All studies defined SVD as intrinsic failure of the bioprosthetic valve, although the specific criteria and diagnostic methodologies varied (Table 3, Supplemental Material 4).

**Table 1:** Summary of data Extracted, including NOS ROB

Study (year)	Population data (patients)	Timepoints (years)	Key findings on SVD	NOS
**Uchino et al. (2022)** [10]	240 (125 St. Jude Medical Epic bioprosthesis^*^, 115 Carpentier-Edwards Perimount Pericardial valve^†^)	3, 5, 7	**Bovine** > Porcine	Low ROB
**Han et al. (2022)** [18]	388 (171 Medtronic Hancock II porcine valves^*^, St. Jude Medical Epic bioprosthesis^*^, St. Jude Biocor^*^, 217 Edwards Lifesciences Perimount^†^, Edwards Lifesciences^†^, Edwards Lifesciences Magna Mitral Ease pericardial valves^†^)	3.5	No significant difference	Low ROB
**Pelletier et al. (1989)** [11]	678 (419 porcine: Standard Carpentier-Edwards Porcine Bioprosthesis^*^, Improved-Annulus Carpentier-Edwards Porcine Bioprosthesis^*^, Supraannular Models Carpentier-Edwards Porcine Bioprosthesis^*^, and 259 bovine: Ionescu-Shiley pericardial bioprosthesis^†^, Carpentier-Edwards pericardial bioprosthesis^†^, Mitroflow valves pericardial bioprosthesis^†^)	6, 10	**Porcine** > Bovine	Low ROB
**Jamieson et al. (1999)** [12]	1695 (1,266 Supraannular Models Carpentier-Edwards Porcine Bioprosthesis^*^, 429 Carpentier-Edwards Perimount Pericardial valve^*^)	5, 10	**Bovine** > Porcine	Low ROB
**Raman et al. (2020)** [13]	274 (56 St. Jude Biocor^*^, 64 Medtronic Hancock II porcine valves^*^, 154 Carpentier-Edwards Perimount Pericardial valve^†^)	10	**Porcine >** Bovine	Low ROB
**Zwischenberger et al. (2024)** [15]	1,162 patients (612 Edwards Lifesciences model 6625 (Carpentier-Edwards) porcine prosthesis^*^, 550 Edwards Lifesciences Model 6900 (Perimount) or 7300 TFX (Magna) pericardial prosthesis^†^)	10	No significant difference	Low ROB
**Kim et al. (2021)** [17]	309 patients (241 Carpentier-Edwards Perimount Pericardial valve^†^, 68 Medtronic Hancock II porcine valves^*^)	5, 10, 15	No significant difference	Moderate ROB
**Grunkemeier et al. (2012)** [16]	344 (181 Carpentier-Edwards porcine valves^*^ and 163 Carpentier-Edwards pericardial bioprosthesis^†^)	8, 15	No significant difference	Moderate ROB
**Beute et al. (2020)** [14]	940 implants (463 Mosaic porcine valve^*^, 477 Carpentier-Edwards Perimount Pericardial valve^†^)	5, 10, 15	**Porcine >** Bovine	Low ROB

SVD, Structural Valve Deterioration; NOS, Newcastle Ottawa Scale; ROB, Risk of Bias

* indicates a porcine valve

† indicates a bovine valve

**Table 2:** Included studies’ designs and affiliations

Study	Institution type	Era compared	Adjustment methods for baseline imbalance
Uchino 2022	Single-center (Hyogo Brain and Heart Center, at Himeji Japan)	Concurrent	Inverse probability weighting
Han 2022	Single-center (Asan Medical Center, Korea)	Concurrent	Propensity score matching
Pelletier 1989	Single-center (Institut de Cardiologie de Montreal, Montreal, Quebec, Canada)	Concurrent	None reported
Jamieson 1999	Multi-center (Canada)	Concurrent	Cox regression analysis
Raman 2020	Single-center (Cleveland Clinic)	Concurrent	Propensity random sampling matching
Zwischenberger 2024	Single-center (Duke University, Durham, North Carolina, USA)	Concurrent	Cox proportional hazards analysis
Kim 2021	Single-center (Seoul National University Hospital)	Concurrent	Propensity score–adjusted multivariable analysis
Grunkemeier 2012	Single health system (Providence/St. Vincent, Oregon)	Different eras (porcine 1974–1996 vs. bovine 1991–2002)	Competing risks regression with covariates
Beute 2020	Single-center (Spectrum Health, Michigan)	Concurrent	Propensity score matching

**Table 3:** Included studies’ definition criterions of SVD

Study	Criterion for definition of SVD
**Uchino et al. (2022)** **Han et al. (2022)** **Pelletier et al. (1989)** **Grunkemeier (2012)** ^*^	Studies created their own criterion
**Jamieson (1999)** ^*^	Edmunds Jr et al. 1996
**Zwischenberger (2024)**	Dvir et al. 2018
**Kim et al. (2021)**	Akins et al. 2008, Baumgartner et al. 2017, and Zoghbi et al. 2009
**Beute et al. (2020)** **Raman et al. (2020)**	Akins et al. 2008

^*^Studies that required the bioprosthetic valve explanted for SVD to be identified

All included articles were retrospective cohort studies, although baseline comparability varied by study design (Table 2). Seven studies were concurrent single-center series [10, 11, 13–15, 17, 18], but Jamieson 1999 was concurrent multi-center series [12], and Grunkemeier 2012 was a single-institution era-separated series [16]. To address baseline imbalances, authors employed inverse probability weighting [10], propensity score matching [13, 14, 18], propensity score–adjusted multivariable analysis [17], Cox models [12, 15], or competing-risks regression with covariates [16]. Pelletier (1989) did not report covariate adjustment [11]. However, multi-center and era-separated designs may still introduce residual confounding from differences in institutional practices, surgical teams, and patient selection despite these adjustment methods.

Two studies [10, 12] favored bovine MVR while three studies [11, 13, 14] favored porcine MVR, with the remainder reporting no significant difference between the valve types [15–18]. All studies were assessed as having a low risk of bias according to the NOS, except for Grunkemeier et al. (2012) [16] and Kim et al. (2021) [17], which scored moderate ROB (Supplemental Material 3).

## Definitions of structural valve deterioration

SVD was the primary outcome in this systematic review, although definitions of SVD varied across the included studies (Table 3, Supplemental Material 4). All studies agreed that SVD involves intrinsic structural or material failure of the bioprosthetic valve that resulted in hemodynamic disruption. However, specific criteria differed; while most studies excluded valve thrombosis, infective endocarditis, and pannus formation from their definition of SVD, Uchino et al. (2022) included pannus formation [10]. More significantly, some studies defined SVD in terms of outcomes [12, 16]; namely, SVD was defined as intrinsic deterioration of the bioprosthetic valve that necessitated reoperation. Methods for diagnosing SVD also varied: some studies relied initially on echocardiographic parameters and/or other clinical indicators, with subsequent confirmation by valve explantation [10, 11, 13, 15, 17, 18], whereas others based the diagnosis exclusively on valve explant findings [12, 16].

### Early SVD outcomes (Up to 8 Years)

Early outcomes provide essential insight into the pivotal years following MVR, as demonstrated by Uchino et al. (2022), who found that the time to SVD ranged from 4.15 to 11.0 years (5.83 ± 1.68 years in the porcine group and 7.29 ± 3.73 years in the bovine group; *p* = 0.107), and Raman et al. (2020), who also found that the mean time to SVD was 8.2 ± 1.1 years in the porcine group and 7.1 ± 0.4 years in the bovine group [10, 13]. Furthermore, Beute et al. (2020) reported an overall average time to reoperation for structural valve deterioration for porcine valves at 11.1 ± 2.3 years and 6.8 ± 2.3 years for bovine valves (*p* < 0.001) [14]. Early SVD findings are summarized in Table 4.

**Table 4:** Early SVD outcomes (Up to 8 Years)

Author (year)	Time point(s)	Result measure	*n* (Porcine/Bovine)	Result
Han et al. (2022)	Median follow-up ~ 3.5 yrs	HR for SVD	171/217	HR = 5.01 (95% CI, 0.65–38.8), *p* = 0.12
Beute et al. (2020)	5 yrs	Freedom from SVD (echocardiography, % [95% CI])	*N* = 940 (not split reported)	Porcine: 91.0% (86.3–94.1); Bovine: 90.3% (85.4–93.6); no significant difference
Uchino et al. (2022)	3, 5, 7 yrs	Freedom from SVD (%)	125/115	Porcine: 97.7%, 90.5%, 75.5%; Bovine: 100%, 96.1%, 90.0%; log-rank *p* = 0.170 after IPW
Jamieson et al. (1999)	5 yrs	Freedom from SVD (%)	638/326	No significant difference
Pelletier et al. (1989)	6 yrs	Freedom from SVD (%)	419/259	Porcine: 92% ± 2%; Bovine: 68% ± 11%; *p* < 0.001
Pelletier et al. (1989)	6 yrs	Valve survival (%)	878/715	Porcine: 80% ± 2%; Bovine: 62% ± 10%; *p* < 0.01
Uchino et al. (2022)	3, 5, 7 yrs	Freedom from SVD reoperation (%)	7/6	Porcine: 98.8%, 95.5%, 81.0%; Bovine: 100%, 98.7%, 95.1%; log-rank *p* = 0.045 after IPW
Grunkemeier et al. (2012)	8 yrs	Probability of explant for SVD (%)	—/163	Bovine: 5% ± 3.2%; no porcine data reported

At a median follow up of ~ 3.5 years, Han et al. (2022) reported some of the earliest data points included in our study [18]. They found no statistically significant difference in the cumulative risks of the development of SVD between bovine (*n* = 217) and porcine (*n* = 171) MVR (HR = 5.01; 95% CI, 0.65–38.8; *p* = 0.12). Their results confirmed what has been shown in pathological studies [7, 12, 14, 15], that reoperations for calcified thickening were more common in the bovine group, whereas leaflet tears or perforations occurred exclusively in porcine MVR patients. At 5 years, Beute et al. (2020) found no difference between echocardiographic freedom from SVD between porcine and bovine MVR (91.0 (86.3–94.1) vs. (90.3 (85.4–93.6) at 95% confidence; *N* = 940) [14]. Uchino et al. (2022) also found no significant difference in the rates of freedom from SVD between groups at 3, 5 and 7 years (97.7%, 90.5% and 75.5% in the porcine group (*n* = 125) and 100%, 96.1% and 90.0% in the bovine group (*n* = 115), respectively) (log-rank *p* = 0.170 after inverse probability of treatment weighted adjustment (IPW)) [10]. Jamieson et al. (1999) agreed by finding no difference in 5-year freedom from SVD between bovine (*n* = 326) and porcine (*n* = 638) [12].

However, after following up for 6 years, Pelletier et al. (1989) demonstrated higher freedom from SVD in porcine valves (92% ± 2%; *n* = 419) compared to bovine valves (68% ± 11%, *n* = 259; *p* < 0.001) [11]. They also noted that significantly more porcine valves survived than bovine pericardial bioprostheses at 6 years (80% ± 2% versus 62% ± 10%; *p* < 0.01). However, with respect to the rate of freedom from SVD reoperation, Uchino et al. (2022) favored the bovine group, reporting significantly higher rates in the bovine group at 3, 5 and 7 years (98.8%, 95.5% and 81.0% in the porcine group (*n* = 7) and 100.0%, 98.7% and 95.1% in the bovine group (*n* = 6), respectively; log-rank *p* = 0.045 after IPW) [10]. As with Han et al. (2022), they also observed that bovine SVD was primarily due to leaflet calcification and adhesion to the subvalvular apparatus, whereas porcine SVD mainly resulted from leaflet tearing or dehiscence, leading to acute mitral regurgitation and an urgent clinical status [10, 18]. Finally, at 8 years, Grunkemeier et al. (2012) also noted that probabilities of explant for SVD were 5% ± 3.2% for bovine MVR (*n* = 163), but did not report comparable data in the porcine position at this time point [16].

## Intermediate SVD outcomes (10-Year Follow-Up)

Intermediate SVD outcomes are summarized in Table 5. At 10 years follow up, Pelletier et al. (1989) reported freedom from primary tissue failure in porcine valves at 54% +/- 6% (*n* = 419), though they did not have any data for bovine valves to compare it to [11]. Raman et al. (2020) also found that actuarial freedom from SVD was higher in the porcine group (96.4 ± 0.08%; CI 94–96%; *n* = 2) than in the bovine group (90.6 ± 0.09%; CI 96–98%; *n* = 9; *p* < 0.05) due to bovine MVR undergoing calcification in 55% of cases, leaflet tear in 33% and mixed aetiology in 12% [13]. Similarly, Beute et al. (2020) reported that the overall cumulative incidence for reoperation was 8.6% (CI 3.6–16.1) for porcine MVR (*n* = 124) and 21.7% (CI 13.7–31.0) in bovine MVR (*n* = 139) (HR = 2.00 (CI 1.05–3.84); *p* = 0.023) [14].

**Table 5:** Intermediate SVD outcomes (10-Year Follow-Up)

Author (year)	Time point(s)	Result measure	*n* (Porcine/Bovine)	Result
Pelletier et al. (1989)	10 yrs	Freedom from primary tissue failure (%)	419/259	Porcine: 54% ± 6%; no bovine data reported
Raman et al. (2020)	10 yrs	Actuarial freedom from SVD (%)	2/9	Porcine: 96.4% ± 0.08% (CI 94–96%); Bovine: 90.6% ± 0.09% (CI 96–98%); *p* < 0.05.
Beute et al. (2020)	10 yrs	Cumulative incidence of reoperation for SVD (%)	124/139	Porcine: 8.6% (CI 3.6–16.1); Bovine: 21.7% (CI 13.7–31.0); HR = 2.00 (CI 1.05–3.84); *p* = 0.023
Jamieson et al. (1999)	10 yrs	Freedom from SVD (%) (age < 70 yrs)	1,266/429	Bovine higher than porcine; *p* = 0.0001
Beute et al. (2020)	10 yrs	Cumulative incidence of reoperation for SVD (%) in patients < 65 yrs	124/139	Porcine: 5.7% (CI 1.8–12.9%); Bovine: 18.8% (CI 11.2–27.9%); *p* = 0.009. Freedom from reoperation: Porcine 94.2% (CI 85.3–97.8%); Bovine 79.0% (CI 67.8–86.7%)
Jamieson et al. (1999)	10 yrs	Freedom from SVD (%) (age > 70 yrs)	34/9	Bovine: 100%; Porcine: 92%
Zwischenberger et al. (2024)	10 yrs	Incidence of structural deterioration (%)	106/50	Porcine: 18% ± 2%; Bovine: 19% ± 3%; *p* = 0.3. Failure modes: severe MS more likely in bovine (70% vs. 36%; *p* < 0.001), severe MR more likely in porcine (75% vs. 38%; *p* < 0.0001). Structural deterioration associated with younger patients (*p* < 0.001), not valve type (*p* = 0.6) or size (*p* = 0.3)

In contrast to the above studies, Jamieson et al. (1999) found that the 10-year freedom from SVD was actually higher for bovine valves (*n* = 429) compared to porcine valves (*n* = 1,266) across all age groups under 70 years (*P* = 0.0001) [12]. In contrast, Beute et al. (2020) found that patients younger than 65 years had a cumulative incidence of reoperation due to solely SVD at 5.7% (95% CI 1.8%−12.9%) for porcine mitral valves (*N* = 124) and 18.8% (95% CI 11.2%−27.9%) for bovine pericardial mitral valves (*N* = 139; *p* = 0.009). Hence, the freedom from reoperative SVD probabilities for those younger than 65 years was 94.2% (95% CI 85.3%−97.8%) for porcine valves and 79.0% (95% CI 67.8%−86.7%) for bovine valves [14].

No significant differences for freedom from SVD were observed by Jamieson et al. (1999) in the age group older than 70 years, but the trend was notable: 100% for bovine bioprostheses (*n* = 9) and 92% for porcine bioprostheses (*n* = 34) at 10 years [12]. They also described the modes of failure for each valve type. Dystrophic calcification only occurred in 70.4% of bovine and 16.9% of porcine prostheses, and leaflet tear without calcification in 18.5% and 26.6%, respectively. The combination of calcification and leaflet tear occurred in 11.1% of bovine and 56.5% of porcine prostheses.

Finally, a recent study by Zwischenberger et al. (2024) showed that the incidence of structural deterioration in porcine (*n* = 106) and bovine MVR (*n* = 50) were similar (18% ± 2% vs. 19% ± 3%, respectively; *p* = 0.3) [15]. The structural failure mode was more likely severe mitral stenosis in bovine than porcine MVR (70% vs. 36%; *p* < 0.001), while it was more likely severe mitral regurgitation in porcine than bovine valves (75% vs. 38%; *p* < 0.0001). Interestingly, after stratifying by age, structural deterioration was associated with younger patients (*p* < 0.001) but not valve type (*p* = 0.6) or valve size (*p* = 0.3) [15].

## Late SVD outcomes (15-Year Follow-Up)

Late SVD outcomes are summarized in Table 6. With respect to 15-year freedom from reoperation due to SVD, Grunkemeier et al. (2012) note that probabilities of explant for SVD were 16% ± 3.3% for porcine MVR (*n* = 181), but did not report outcomes for bovine MVR at 15 years [16]. Beute et al. (2020) reported that for reoperation due to SVD alone, the overall cumulative incidence was 2.32 (95% CI 1.31–4.11) times higher in the bovine valves than the porcine valves, with a cumulative incidence of SVD at 13.2% (95% CI 8.1%−19.5%) for bovine valves (*n* = 401) and 7.9% (95% CI 4.7%−12.3%) for porcine valves (*n* = 401; *p* < 0.001)) [14]. Consequently, freedom from reoperative probabilities for SVD was 92.3% (95% CI 87.6%−95.3%) for porcine valves and 86.7% (95% CI 79.4%−91.6%) for bovine valves [14].

**Table 6:** Late SVD outcomes (15-Year Follow-Up)

Author (year)	Time point(s)	Result measure	*n* (Porcine/Bovine)	Result
Grunkemeier et al. (2012)	15 yrs	Probability of explant for SVD (%)	181/—	Porcine: 16% ± 3.3%; bovine data not reported
Beute et al. (2020)	15 yrs	Cumulative incidence of reoperation for SVD (%)	401/401	Porcine: 7.9% (95% CI 4.7–12.3%); Bovine: 13.2% (95% CI 8.1–19.5%); *p* < 0.001; HR = 2.32 (95% CI 1.31–4.11)
Beute et al. (2020)	15 yrs	Freedom from reoperation for SVD (%)	401/401	Porcine: 92.3% (95% CI 87.6–95.3%); Bovine: 86.7% (95% CI 79.4–91.6%)
Beute et al. (2020)	15 yrs (< 65y)	Cumulative incidence of reoperation for SVD (%) in patients < 65 yrs	124/139	Porcine: 15.8% (95% CI 7.4–27.0%); Bovine: 30.2% (95% CI 15.1–46.8%); *p* = 0.009
Beute et al. (2020)	15 yrs (< 65y)	Freedom from reoperation for SVD (%) in patients < 65 yrs	124/139	Porcine: 84.0% (95% CI 70.9–91.6%); Bovine: 68.1% (95% CI 46.4–82.5%)

Interestingly, in patients younger than 65 years, cumulative incidence of reoperation due to solely SVD was 15.8% (95% CI 7.4%−27.0%) for porcine mitral valves (*N* = 124) and 30.2% (95% CI 15.1%−46.8%) for bovine pericardial mitral valves (*N* = 139; *p* = 0.009) [14]. As a result, the freedom from reoperative SVD probabilities for those younger than 65 years was 84.0% (95% CI 70.9%−91.6%) for porcine valves and 68.1% (95% CI 46.4%−82.5%) for bovine valves [14]. SVD by stenosis was observed in 96% of patients in the bovine cohort, but only 37% in the porcine cohort [14]. The primary mode of failure for porcine valves was calcification of the leaflets (50%), with regurgitation usually from leaflet separation at the annulus [14].

### Cumulative findings across Follow-Up timepoints

Cumulative SVD outcomes across 5-, 10-, and 15-year follow-up periods are summarized in Table 7. Overall, Beute et al. (2020) found that porcine valves had a statistically significant better freedom from both operative and nonoperative SVD compared with bovine valves over 5, 10, and 15 year timepoints (*N* = 802; *p* = 0.002) [14]. However, Kim et al. (2021) also reported cumulative incidences of SVD at 5, 10, and 15 years, but did not find a significant difference between bovine and porcine groups (32.4% (*n* = 33) vs. 41.7% (*n* = 17), respectively; *p* = 0.23) [17].

**Table 7:** Cumulative findings across Follow-Up timepoints

Author (year)	Time point(s)	Result measure	*n* (Porcine/Bovine)	Result
Beute et al. (2020)	5, 10, 15 yrs	Freedom from operative + nonoperative SVD (%)	401/401	Porcine significantly better than bovine; *p* = 0.002
Kim et al. (2021)	5, 10, 15 yrs	Cumulative incidence of SVD (%)	17/33	Porcine: 41.7%; Bovine: 32.4%; *p* = 0.23; no significant difference

## Discussion

Our systematic review provides a comprehensive evaluation of SVD in MVR using bovine pericardial and porcine bioprosthetic valves. By synthesizing data from 9 studies involving 6,945 patients with follow-up periods ranging from 3.5 to 15 years, our analysis offers critical insights into the long-term durability and modes of failure of these valve types. The findings underscore the complex interplay between valve tissue characteristics, patient demographics, and longitudinal effects, highlighting important considerations for clinical decision-making.

### Key findings

Continuous assessment for SVD after MVR could be recommended, since the time to SVD ranged from 3.5 to 13 years [10, 13, 14]. Notably, porcine valves may demonstrate a longer onset to SVD compared to bovine valves [10, 13, 14]. Early outcomes did not find significant differences between bovine and porcine MVRs until after 5 years (*N* = 2532) [10, 12, 14, 18]. Pelletier et al. (1989) provided strong evidence to support porcine valves in terms of freedom from SVD and MVR survival in the post-five year period (*N* = 678) [11]. Although the results from Uchino et al. (2022) contradicted what was found in the study by Pelletier et al. [10, 11], their study population was marginal in comparison to Pelletier et al. (1989) (*N* = 13 vs. *N* = 678, respectively) [11]. Grunkemeier et al. (2012) reported data for bovine MVR at 8 years, but did not make any comparison with porcine MVR at this time point [16]. Given that they later reported outcomes for porcine MVR at 15 years, it is likely that comparable 8-year data were available but not reported, suggesting a potential reporting bias (Table 1; Supplemental Material 3).

Intermediate SVD outcomes comparing bovine and porcine MVRs were split between studies, with some favoring bovine (*N* = 1,695) and other porcine (*N* = 274) [13, 14]. Interestingly, Zwischenberger et al. (2024) did not find a difference between the valve types (*N* = 156), but after stratifying by age, they found a higher incidence of SVD with younger patients [15]. This may be due to increased cyclical loading or stronger immune responses accelerating calcifications [24]. However, Jamieson et al. (1999) (*N* = 1,695) and Beute et al. (2020) (*N* = 263) disagreed on whether bovine or porcine valves were better for younger patients, respectively, highlighting the need not only for continued research in this area, but also that age may be an effect modifier in MVR [12, 14].

Finally at 15 years follow up, Beute et al. (2020) was the only study that made direct comparisons between bovine and porcine MVR, strongly favoring porcine valves (*n* = 32) over bovine valves (*n* = 53) [14]. Grunkemeier et al. (2012) used competing risk regression (CRR) to predict their bovine data at 8 years follow up to 15 years [16]. Then they compared this to their actual 15 year follow up data for porcine valves, and found no significant difference between the two MVR types. Perhaps due to small sample size, Kim et al. (2021) did not make direct comparisons between bovine (*n* = 3) and porcine (*n* = 3) MVRs at 15 years despite having the data. Instead they reported cumulative incidences between groups (*N* = 50), and found no difference [17]. However when Beute et al. (2020) analyzed their cumulative data (*N* = 802), they found greater freedom from SVD in the porcine group [14].

In short, the compiled evidence from these nine studies indicates that while both bovine and porcine bioprosthetic mitral valves exhibit acceptable mid- to long-term durability, differences in the pattern and timing of SVD are evident. Although two studies favored bovine MVR and three favored porcine MVR, four studies did not find any difference.

### Structural valve deterioration definitions

A universally accepted definition for SVD remains elusive, complicating reliable comparisons of outcomes in the literature on bioprosthetic MVR. Earlier consensus efforts introduced by Edmunds et al. (1996) [25] and Akins et al. (2008) [26] were utilised by several studies in our systematic review (Supplemental 4). More recently however, the American College of Cardiology (ACC) and American Heart Association (AHA) 2020 Valvular Heart Disease Management Guidelines [8] recognize the 2018 study by Dvir et al. [5] as a promising framework for standardizing SVD definitions in MVR, which was utilized in the recent study by Zwischenberger et al. (2024) [15]. Despite these advances, many studies [10, 11, 16, 18] continue to employ varied definitions, some creating their own definitions or combining previously established definitions [27, 28], underscoring the urgent need for universal standardization.

### SVD echocardiographic parameters

Several cohorts used a hybrid approach, counting SVD when doppler criteria were crossed and then confirming the diagnosis if the valve was eventually explanted [10, 11, 13–15, 17, 18]. Yet the Doppler thresholds they chose were not uniform. Uchino (2022) [10], Zwischenberger (2024) [15], and Beute (2020) [14] labelled SVD once the transmitral mean gradient exceeded 10 mm Hg or regurgitation reached a severe grade, whereas Kim (2021) [17] used a 6 mm Hg gradient and accepted moderate-or-worse regurgitation. Sensitive cut-offs (e.g., Kim (2021) [17]) capture milder lesions which theoretically should introduce earlier and more frequent SVD “events”. Yet, short-, mid-, and long-term data across these cohorts show a different story.

Short-term (5 years): Kim (2021) reported < 4% SVD cumulative incidence [17]. This was slightly greater than the stricter Uchino (2022) and Beute (2020) series, which stayed >90% through the same interval [10, 14].

Mid-term (10 years): Kim (2021) and Zwischenberger (2024) reported similar cumulative SVD incidence of < 20% for both valve types [15, 17]. Beute (2020) had a 10-year freedom from SVD of 76.8% for porcine and 54.6% for bovine (Uchino (2022) did not provide a 10-year estimate) [10, 14].

Long-term (15 years): Kim (2021) reported 32.4% cumulative SVD incidence for bovine and 41.7% for porcine [17]. Beute (2020) reported a freedom of SVD of 26.2% for bovine and 33.4% for porcine (Zwischenberger (2024) did not report 15-year SVD data) [14, 15].

While short-term data (5 years) shows a broadly similar freedom from SVD regardless of doppler threshold, 10 year and 15 year data show some discrepancy, particularly Beute who surprisingly had a stricter doppler threshold. This difference may be driven by the differing valve model types in the study.

Other included studies utilise purely qualitative data for SVD detection by echocardiography (i.e., Pelletier (1989) and Raman (2020)) [11, 13]. Still, the aforementioned studies align conceptually with the other studies in that SVD is intrinsic leaflet failure seen on echo. Interestingly, in mid-term data (10 years), freedom from SVD for Pelletier (1989) was much lower than Kim (2021) in both valve types, whereas Beute (2020) had a much higher freedom from SVD at 10 years for both valve types, although both defined qualitative echo parameters. This could be because Pelletier utilized older valve models compared to Raman.

In short, all the included studies seek to capture intrinsic leaflet deterioration on imaging. Kim (2021) adopts a more sensitive Doppler threshold, Uchino (2022), Zwischenberger (2024), and Beute (2020) hew to more specific criteria, and Pelletier (1989)/Raman (2020) remain mechanism-based without prescribing exact numbers. Differences in freedom from SVD are more likely due to valve models and differences in data analysis than echo parameters per se.

### Modes of SVD

The failure mode of porcine versus bovine valves is well-studied. In particular, porcine valves tend to fail due to regurgitation, whereas bovine valves tend to fail due to stenosis [7, 12, 14, 15]. The underlying pathological process in both cases is most likely due to glutaraldehyde treatment of the valves, which decreases immunogenicity at the cost of increasing calcification [29]. In the porcine valves, the calcification leads to leaflet tearing and consequent regurgitation, while in the bovine valves, dystrophic calcification causes stenosis [12]. The exact mechanism of calcifications in mitral prostheses appears to be similar to aortic prostheses, though valves in the mitral position are at higher risk for calcification, likely due to higher mechanical stress on the closed leaflets during ventricular systole [30]. Calcification typically begins in the area of leaflet flexion in both bovine and porcine prostheses [31]; however, in porcine prostheses, calcium tends to deposit earlier on collagen cords and create focal stiffness, which causes adjacent tissue to bear more stress and become more susceptible to microtears [32], which can propagate and lead to leaflet tearing. However, other studies have proposed that the mechanism of leaflet tears is due to accumulated mechanical damage independent of calcification [33]. In bovine valves, although calcification begins in the area of leaflet flexion, it is more diffuse than in porcine valves, likely owing to increased rigidity (which is less prone to tearing) [7]. The diffuse calcification then leads to diffuse stiffness, which is the cause of stenosis. While these patterns hold at the population level, there are also a plethora of host factors that predispose to calcification, including the immune/inflammatory response, oxidative stress, metabolic disorders, and subclinical thrombosis [34].

Porcine devices such as the St. Jude Medical Epic and St. Jude Biocor valves tended to develop leaflet tears as a consequence of structural deterioration, manifesting clinically with acute or progressive mitral regurgitation. In Han et al. (2022), all the valve tears occurred in the porcine valves, namely Epic and Biocor [18]. In Uchino et al. (2022), six of the seven reoperations in the Epic group were due to leaflet tearing, all of which were causes of acute heart failure [10]. Meanwhile, other porcine lines, such as the Medtronic Hancock II and Mosaic valves, were studied by Han et al. (2022), Raman et al. (2020), Kim et al. (2021), and Beute et al. (2020); these likewise predominantly failed through tears [13, 14, 17, 18]. These findings agree with pathological examinations of modes of porcine mitral valve failure, which note that these valves tend to fail due to tears secondary to higher stress at stent posts [35].

By contrast, the various Carpentier-Edwards pericardial valves, often collectively referred to as Perimount (Carpentier-Edwards Perimount Pericardial valve and Magna), have historically presented with gradual leaflet thickening and calcific deposits leading to mitral stenosis. For instance, Jamieson et al. (1999) and Kim et al. (2021) described the progression of steno‐insufficiency in their pericardial cohorts with CEPP [12, 17]. Uchino et al. (2022), in contrast to its findings on Epic porcine valves, observed subvalvular adhesions and calcification in its CEPP bovine‐valve patients, typically unfolding more gradually and requiring reoperation at a much later date after diagnosis of SVD compared to the acute reoperation needed in the Epic porcine valves [10]. Among earlier models, the Ionescu‐Shiley pericardial valve showed particularly accelerated degeneration in the mitral position, as reported by Pelletier et al. (1989), who found that the majority of pericardial‐valve reoperations in their cohort arose from that design [11].

Contemporary bioprostheses incorporate advanced anticalcification treatments. Specific agents include glutaraldehyde and alpha-amino oleic acid for the Mosaic porcine valve [14], XenoLogix for the Carpentier-Edwards Perimount [13], and Thermafix for Edwards Magna [14]. Others included sodium tetradecyl sulphate for the Hancock II valve [13], polysorbate 80 in the Carpentier-Edwards supra-annular porcine and Perimount pericardial valves [12], and RESILIA™ tissue treated with ethanol rinsing and glycerolization in bovine pericardial valves [17].

Additional or alternative treatments are being investigated for use with both bovine and porcine valves. For instance, Meuris et al. (2018) note that octanediol-ethanol based phospholipid removal with taurine-based glutaraldehyde neutralization and storage in an aldehyde-free solution reduced both phospholipid levels and calcification levels in rats [36], while Jannasch et al. (2024) show that a glutaraldehyde-free approach combining decellularization, riboflavin/UVA-cross-linking, and low-energy electron beam irradiation achieves similar stiffness with less calcification in vivo [37].

### Length time bias

Grunkemeier et al. (2012) and Jamieson et al. (1999) both described SVD using evidence on explant only, whereas other studies utilised both echocardiographic imaging and explantation, thus producing significant heterogeneity in the degree of SVD reported [12, 16].

In the explant-based study by Jamieson et al. (1999), actuarial freedom from SVD in patients aged 61–70 years remained relatively high throughout the first decade; 100% for bovine versus 98.4% for porcine valves at five years, and 95.2% versus 75.2% at ten years [12]. Grunkemeier et al. (2012) shows a similarly slow attrition when SVD is counted only at surgery: SVD incidence was 5% ± 3.2% for bovine prostheses at eight years (5 or 10 year year data was not mentioned) [16]. While Jamesison lacks long-term outcomes (15 years), the porcine prostheses in Grukenmier (2012) showed a SVD incidence of 16% ± 3.3%.

Echocardiographic surveillance paints a much earlier descent. Beute et al. (2020)’s study showed an echo-defined freedom of 91.0% (porcine) versus 90.3% (bovine) at five years, 76.8% versus 54.6% at ten years, and only 33.4% versus 26.2% by fifteen years [12]. Yet in the same series, when the analysis was limited to re-operation for SVD alone, freedom from reoperative SVD at fifteen years was 92.3% for porcine prostheses, similar to the explant-based SVD numbers reported by Grunkemeier et al. (2012) [12].

Taken together, these contrasts illustrate length time bias. Routine imaging detects many slowly progressive lesions years before they necessitate explantation, whereas explant-based definitions record SVD only at its end-stage presentation. Consequently, explant-based freedom from SVD curves appear flatter and shift failure into the second decade, whereas echo-based curves begin to decline within the first decade despite describing the same biological process.

### Strengths

In comparing our review with previous systematic reviews, it is clear that our approach offers several distinct advantages. Earlier reviews often combined studies that reported SVD outcomes for bovine or porcine valves independently, then drew indirect comparisons. For instance, while the 2019 meta-analysis by Malvindi et al. and the 2024 meta-analysis by Koulouroudias et al. provided valuable insights, they were limited by non-uniform SVD definitions, reliance on reconstructed patient data, and indirect comparisons. In contrast, our review exclusively includes studies that directly compare bovine versus porcine valves in the mitral position. This focus theoretically attempts to reduce confounding factors such as variations in surgical techniques, surgeon expertise, and patient demographics, thereby potentially offering a more reliable assessment of valve performance.

### Limitations

Despite the strengths of our methodology, several challenges emerged during our review that also highlight the inherent limitations of the current literature. There was considerable variability in how studies reported their results; key outcome parameters such as SVD definitions, specific numerical results, confidence intervals, p‑values, and follow‑up durations were not standardized. Some studies provided detailed statistical data, while others offered only limited or ambiguous information. Furthermore, follow‑up periods ranged widely from as little as 3.5 years to as long as 15 years and many reports lacked long‑term follow-up data.

Although only studies with direct head-to-head comparisons were included, baseline characteristics were not necessarily uniformly balanced across all studies. Multi-center registries and era-separated comparisons introduce potential confounding from differences in institutional practices and patient selection. Not to mention that even in single-center studies, valve choice would have been at surgeon discretion. While most contemporary series used propensity score matching, propensity score–adjusted multivariable analysis, Cox regression analysis, Cox proportional hazards analysis, inverse probability weighting, or competing-risks regression to mitigate baseline imbalances, residual confounding remains possible.

We also acknowledge that structural valve deterioration can be influenced by multiple interacting variables, including patient characteristics, valve design, and hemodynamic performance. For example, because most included studies compared a specific porcine model with a specific bovine model, the observed differences may reflect model-specific performance rather than intrinsic differences between xenograft materials. Model-specific design features such as valve geometry, leaflet mounting configuration, and stent design could all contribute to structural valve deterioration, rather than xenograft material alone.

Another important limitation is the evolution of valve manufacturing processes across the long time span of the included studies (1989–2024). Early-generation prostheses, such as the Ionescu–Shiley pericardial valves examined in Pelletier (1989) [11], lacked contemporary anticalcification treatments, whereas more recent bovine valves such as the Edwards Perimount Magna incorporate proprietary processes such as *Thermafix* [13, 14]. Likewise, modern porcine devices such as the Medtronic Mosaic use phospholipid extraction technologies that were not available in earlier Hancock models [14]. These advancements are known to reduce leaflet calcification and may therefore improve durability, potentially influencing the differences in structural valve deterioration observed between studies and across valve types [15]. However, because manufacturers rarely disclose complete details of their anticalcification protocols, and because no study stratified outcomes by valve generation or treatment process, we were unable to fully control for this source of heterogeneity. The variation in prosthesis design and treatment across decades should therefore be considered when interpreting the pooled findings.

While an analytical approach such as multivariable or meta-regression modeling would be ideal to isolate these effects, the lack of consistent reporting across studies precluded such an analysis in this review. Additionally, advances in pericardial valve technology may significantly change results in future studies. For example, the MITRIS Resilia is a new bovine pericardial valve that incorporates Resilia tissue into the valve to increase durability [38]. The Dafodil Neo is another relatively new valve with stenting, with early studies indicating good performance with respect to SVD [39].

SVD definitions were a notable limitation due to their lack of uniformity. Less ambiguous end points like freedom from reoperation could be considered, however, since the primary aim was to capture the full spectrum of SVD including earlier echocardiographic changes that precede, and may never culminate in, re‑operation, we did not pool re‑intervention data in this review. A focus on reintervention could be considered in subsequent reviews.

Finally, valve grouping in our review followed the classifications used by the primary studies, which directly compared bovine and porcine bioprosthetic valves within their respective generations. Although confounding factors across design eras undoubtedly exist, these were not consistently reported in the source studies, limiting our ability to control for them. Nonetheless, we believe that synthesizing these comparisons provides valuable historical and clinical context for understanding the ongoing conversation of the evolving performance of the two valve types.

### Clinical applications

The clinical implications of our findings are substantial, particularly in the context of personalized patient care. Nuanced differences were observed between bovine and porcine valves, for example, the predominance of calcification leading to stenosis in bovine valves versus leaflet tearing in porcine valves, and differences in the timing of SVD. Notably, SVD from calcification may have a more insidious onset, whereas leaflet tears can present acutely and require emergent intervention [10]. Thus, valve selection should be tailored to the individual patient’s needs. Additionally, cultural and religious considerations are essential; patients from communities that adhere to Jewish, Islamic, or Adventist dietary laws, which restrict the use of porcine products, may prefer bovine-derived valves. Such personalized considerations are crucial for optimizing long-term outcomes and ensuring that patient values are factored into clinical decision-making.

### Call for future studies

Looking forward, our review highlights the urgent need for future studies to overcome the current limitations. While large-scale randomized controlled trials (RCTs) could, in theory, clarify the comparative durability of porcine versus bovine mitral valves, such trials face substantial challenges. Any head-to-head comparison of “porcine” versus “bovine” valves would necessarily compare specific valve models, making it difficult to separate the effects of xenograft material from model design. One possible approach would be a factorial design, in which patients are randomized by both xenograft material and valve model. This could help isolate material effects, but would require extremely large sample sizes, multi-manufacturer participation, and standardized surgical techniques, making it logistically and financially demanding. There is a pressing need for large, multi-center prospective studies, patient-level pooled analyses, and standardized observational designs that can address these confounding issues while still providing high-level comparative evidence. In addition, developing comprehensive patient databases would facilitate patient-level meta-analyses, allowing for more precise comparisons through methods such as propensity-score matching to control for confounding variables.

It is also imperative that future research adopts standardized reporting practices, including consistent outcome measures, follow-up time points, and uniform definitions of SVD. Moreover, the advent of novel treatments for valves may have to be considered in future studies. Given that the desired effect is the reduction of calcification, which will likely reduce both modes of failure, but not necessarily evenly, this could drastically change both short- and long-term results in future studies. This is especially relevant for meta-analyses that might otherwise fail to account for effect modification. As novel valve treatments are made available in bovine and porcine mitral valves, further studies will be required to provide a more accurate estimate of outcomes such as SVD. Furthermore, because the modes of failure may impact long-term results [12], and because novel treatments may not affect the modes of calcification evenly across the valve types, long-term follow-ups should be planned.

While our review focuses on structural valve deterioration, it is important to recognize that valve selection in clinical practice is multifactorial. Other pathological processes, as well as patient comorbidities, surgical factors, and long-term management considerations, also influence outcomes. A future study evaluating overall valve explant or failure rates across various pathological mechanisms could offer a more comprehensive perspective.

Finally, further exploration into re-replacement strategies using percutaneous techniques is warranted, particularly given that calcified bovine valves may interfere with valve-in-valve implantation procedures [40]. By addressing these areas, future research can provide a more accurate estimate of outcomes and ultimately guide more effective clinical decision-making in MVR.

## Conclusion

This systematic review provides a nuanced comparison of bovine and porcine bioprosthetic valves in mitral valve replacement, revealing important differences in structural valve deterioration and reoperation outcomes. By focusing exclusively on studies that directly compared the two valve types, our work addresses a critical gap in the literature and enhances the reliability of the findings. Although variability in reporting standards and follow-up durations remains a challenge, the insights gathered herein underscore the need for personalized valve selection in clinical practice, taking into account factors such as patient demographics and cultural considerations. Moving forward, rigorous, standardized studies and patient-level analyses are essential to further refine our understanding and ultimately improve long-term patient outcomes.

## Supplementary Information

Supplementary Material 1

Supplementary Material 2

Supplementary Material 3

## Data Availability

No datasets were generated or analysed during the current study.

## Abbreviations

SVD: Structural Valve Deterioration
NOS: Newcastle Ottawa Scale
ROB: Risk of Bias
STS: Society of Thoracic Surgery
AATS: American Association for Thoracic Surgery
EACTS: European Association for Cardiothoracic Surgery
AHA: American Heart Association
ACC: American College of Cardiology
EAPCI: European Association of Percutaneous Cardiovascular Interventions
ESC: European Society of Cardiology
