# Supplementary material for: Comparing porcine versus bovine mitral valve replacement in terms of structural valve deterioration: a systematic review
Source: J Cardiothorac Surg. 2025 Nov 26;20:441. doi: 10.1186/s13019-025-03686-2 (PMC12659258; doi:10.1186/s13019-025-03686-2)
Supplement: Supplementary file 1 — Supplementary Material 1 [file 13019_2025_3686_MOESM1_ESM.docx]

**Supplemental Material 1: Search Strings**

**MEDLINE**

1. (MH "Mitral Valve Insufficiency") OR (MH "Mitral Valve Stenosis") OR (MH "Mitral Valve")
2. AB ( “Mitral Valve Disease*” OR “Mitral Valve Replacement*” OR “MVR” OR "mitral valve regurgitation" OR “mitral valve repair”) OR TI (“Mitral Valve Disease*” OR “Mitral Valve Replacement*” OR “MVR” OR "mitral valve regurgitation" OR “mitral valve repair”)
3. S1 OR S2
4. AB ("bovine pericardial valve bioprosthesis" OR "bovine pericardial valve*" OR "bovine bioprosthetic valve*" OR "bovine valve*" OR "cow valve*" OR "Bovine Pericardial Bioprosthetic valve*" OR "porcine pericardial valve bioprosthesis" OR "porcine pericardial valve*" OR "porcine bioprosthetic valve*" OR "porcine valve*" OR "pig valve*" OR "Porcine Pericardial Bioprosthetic valve*") OR TI ("bovine pericardial valve bioprosthesis" OR "bovine pericardial valve*" OR "bovine bioprosthetic valve*" OR "bovine valve*" OR "cow valve*" OR "Bovine Pericardial Bioprosthetic valve*" OR "porcine pericardial valve bioprosthesis" OR "porcine pericardial valve*" OR "porcine bioprosthetic valve*" OR "porcine valve*" OR "pig valve*" OR "Porcine Pericardial Bioprosthetic valve*")
5. S3 AND S4

**EMBASE**

1. 'mitral valve'/exp OR 'mitral valve stenosis'/exp OR 'mitral valve replacement'/exp OR 'mitral valve disease'/exp OR 'mitral valve regurgitation'/exp OR 'mitral valve repair'/exp
2. 'mitral valve insufficiency':ti,ab OR 'mvr':ti,ab
3. #1 OR #2
4. 'bovine pericardial valve bioprosthesis'/exp OR 'porcine pericardial valve bioprosthesis'/exp
5. 'bovine pericardial valve*':ti,ab OR 'bovine bioprosthetic valve*':ti,ab OR 'bovine valve*':ti,ab OR 'cow valve*':ti,ab OR 'bovine pericardial bioprosthetic valve*':ti,ab OR 'porcine pericardial valve*':ti,ab OR 'porcine bioprosthetic valve*':ti,ab OR 'porcine valve*':ti,ab OR 'pig valve*':ti,ab OR 'porcine pericardial bioprosthetic valve*':ti,ab
6. #4 OR #5
7. #3 AND #6

**WEB OF SCIENCE**

1. TS=('mitral valve' OR 'mitral valve stenosis' OR 'mitral valve replacement' OR 'mitral valve disease' OR 'mitral valve regurgitation' OR 'mitral valve repair' OR 'mitral valve insufficiency' OR 'mvr')
2. TS=('porcine pericardial valve*' OR 'porcine bioprosthetic valve*' OR 'porcine valve*' OR 'pig valve*' OR 'porcine pericardial bioprosthetic valve*' OR 'porcine pericardial valve bioprosthesis' OR 'bovine pericardial valve bioprosthesis' OR 'bovine pericardial valve*' OR 'bovine bioprosthetic valve*' OR 'bovine valve*' OR 'cow valve*' OR 'bovine pericardial bioprosthetic valve*')
3. #1 AND #2

**Supplemental Material 2: Inclusion/Exclusion Criteria**

**Inclusion Criteria**

**Population:**

- Adult Patients requiring Mitral Valve Replacement (MVR)

**Intervention:**

- Porcine Bioprosthetic Mitral Valve Replacement - ie, St. Jude Medical Epic bioprosthesis, Medtronic Hancock II porcine valves, Standard Carpentier-Edwards Porcine Bioprosthesis, Improved-Annulus Carpentier-Edwards Porcine Bioprosthesis, Supraannular Models Carpentier-Edwards Porcine Bioprosthesis, Edwards Lifesciences model 6625 (Carpentier-Edwards) porcine prosthesis, Carpentier-Edwards porcine valves, Mosaic porcine valve, St. Jude Biocor

**Comparison:**

- Bovine Pericardial Bioprosthetic Mitral Valve Replacement - ie, Carpentier-Edwards Perimount Pericardial valve, Edwards Lifesciences, Edwards Lifesciences Perimount, Edwards Lifesciences Magna Mitral Ease pericardial valves, Ionescu-Shiley pericardial bioprosthesis, Carpentier-Edwards pericardial bioprosthesis, Mitroflow valves pericardial bioprosthesis, Edwards Lifesciences Model 6900 (Perimount) or 7300 TFX (Magna) pericardial prosthesis

**Outcome:**

- Diagnosis of Structural Valve Deterioration
- Reoperation due to Structural Valve Deterioration;
  - "Structural valve deterioration is any change in function (a decrease of one New York Heart Association functional class or more) of an operated valve resulting from an intrinsic abnormality of the valve that causes stenosis or regurgitation.
  - Wear, fracture, poppet escape, calcification, leaflet tear, stent creep, and suture line disruption of components (eg, leaflets, chordae) of an operated valve early mortality, survival, and freedom from SVD of mitral prostheses.”

**Study Characteristics:**

- Randomized controlled trials or observational studies or cohort (longitudinal) studies or retrospective cohort studies or case-control studies
- Only studies that report direct comparisons between bovine and porcine MVR

**Exclusion Criteria:**

**Population:**

- In vitro or animal studies
- Research on a cadaver
- Paediatric patients
- Cardiac surgery excluding mitral operation

**Intervention:**

- Valve Replacement excluding Porcine Bioprosthetic Mitral Valve Replacement
- Transcatheter valves
- Studies involving Aortic Valve Replacements
- Studies that consolidated data on valve replacements in both aortic and mitral positions
- Double valve replacements using bioprosthetic valves (combined aortic and mitral)
- MVR using mechanical valves

**Comparator:**

- Valve Replacement excluding Bovine Bioprosthetic Mitral Valve Replacement
- Transcatheter valves
- Studies involving Aortic Valve Replacements
- Studies that consolidated data on valve replacements in both aortic and mitral positions
- Double valve replacements using bioprosthetic valves (combined aortic and mitral)
- MVR using mechanical valves

**Outcome:**

- Structural valvular deterioration due to infection or thrombosis as determined by reoperation, autopsy, or clinical investigation.

**Study Characteristics:**

- Editorials, expert opinions, commentaries, or letters to the editor
- Studies that report SVD outcomes for either bovine OR porcine MVR but do not make direct comparisons between the two valve types.

**Supplemental Material 3: Detailed NOS ROB**

<https://docs.google.com/spreadsheets/d/1_gSlqZI55lwZhwYv9EhP5UKwN5eTYk5jbEBhaUbz3-I/edit?usp=sharing>

**Supplemental Material 4: SVD Definition by Study**

| SVD Definition by Study | | |
| --- | --- | --- |
| STUDY | Criterion | Definition |
| Uchino (2022) [(10)](https://www.zotero.org/google-docs/?Cxr5s0) | Study used their own criterion | One of the following on echocardiographic assessment:   1. Severe mitral regurgitation (effective regurgitant orifice area >40 mm2 2. Regurgitant volume >60 ml 3. Visual confirmation of leaflet tearing or prolapse using TTE or TEE 4. Stenosis (transmitral mean pressure gradient > 10 mmHg)   OR:  Those who didn’t meet the above criteria but had:   1. Mitral stenosis and/or Regurgitation 2. Clinical symptoms: (fatigue / dyspnoea / oedema) 3. Suggestive Tricuspid Regurgitation pressure gradient / Pulmonary Regurgitation pressure gradient / Pulmonary Vein flow.   But not:  Prosthetic valve endocarditis, thrombosis, or paravalvular leakage. |
| Han (2022) [(18)](https://www.zotero.org/google-docs/?NwM1YB) | Study used their own criterion | Re-operation due to either one of:   1. Mitral Leaflet tear / perforation 2. Mitral Leaflet thickening / calcification   But not:  Infective endocarditis, pannus formation, or paravalvular leakage. |
| Pelletier (1989) [(11)](https://www.zotero.org/google-docs/?0sjutu) | Study used their own criterion | Mitral Valve Dysfunction with Regurgitation due to one of:   1. Leaflet tear, stretching, or creeping of the stent, 2. Valvular obstruction by leaflet fibrosis / calcification. |
| Jameison (1999)^*^ [(12)](https://www.zotero.org/google-docs/?1r1K9E) | Edmunds et al. 1996 | SVD diagnosed at explant reoperation via:   1. Intrinsic Mitral Valve changes (wear, fracture, poppet escape, calcification, leaflet tear, stent creep, suture line disruption of components)   And   1. Any decrease in function (NYHA) of an operated valve that causes stenosis / regurgitation.   But not deterioration/dysfunction due to infection or thrombosis causes. |
| Raman (2020) [(13)](https://www.zotero.org/google-docs/?faEuQu) | Akins et al. 2008 | Reoperation due to Mitral Valve calcification and/or leaflet tear |
| Zwischenberger (2024) [(15)](https://www.zotero.org/google-docs/?1OtmM2) | Dvir et al. 2018 | One of:   1. Severe mitral regurgitation / stenosis 2. Reoperation for mitral regurgitation / stenosis   But not:  Caused by endocarditis |
| Kim (2021) [(17)](https://www.zotero.org/google-docs/?fIhtLn) | Akins et al. 2008, Baumgartner et al. 2017, and Zoghbi et al. 2009 | Intrinsic xenograft changes (Leaflet thickening, calcification, tear) noted on re-operation and one / more of the following echocardiography findings:   1. Mean pressure gradient > 6 mmHg) 2. Mitral Regurgitation greater / equal to moderate degree (4-6)   But not:  Changes in echocardiographic data due to prosthetic valve endocarditis. |
| Grunkemeier (2012)^*^ [(16)](https://www.zotero.org/google-docs/?fRiFw9) | Study used their own criterion | Explant for SVD and one / more of:   1. Leaflet tears 2. Fibrosis 3. Calcification |
| Beute (2020) [(14)](https://www.zotero.org/google-docs/?IwJdUD) | Akins et al. 2008 | Intrinsic mitral valve changes *(such as wear, fracture, poppet escape, calcification, leaflet tear, stent creep, suture line disruption of components, new chordal rupture, leaflet disruption, or leaflet retraction of a repaired valve)* leading to:  Dysfunction or deterioration involving the operated valve, determined by reoperation, autopsy, or clinical investigation  But not due to:  Infection or thrombosis |

^*^: Studies that required explantation for SVD to be identified.
